# Lumacaftor/ Ivacaftor improves exercise tolerance in patients with Cystic Fibrosis and severe airflow obstruction

**DOI:** 10.1186/s12890-019-0866-y

**Published:** 2019-06-17

**Authors:** Peter A. B. Wark, Kim Cookson, Theeba Thiruchelvam, John Brannan, Douglas J. Dorahy

**Affiliations:** 1 Centre for Healthy Lungs, Hunter Medical Research Institute, University of Newcastle, Lookout Rd New, Lambton, NSW 2305 Australia; 2 Adult Cystic Fibrosis Centre, Department of Respiratory and Sleep Medicine, John Hunter Hospital, Newcastle, Australia

**Keywords:** Cystic fibrosis, Lumacaftor, Ivacaftor, Six minute walk test, Spirometry, Lung transplantation

## Abstract

**Background:**

Treatment of patients with Cystic Fibrosis homozygous for the Phe508del gene, with *Lumacaftor* /*Ivacaftor* (LUM/IVA) improves outcomes in patients with FEV1 > 40% predicted. We set out to observe the most sensitive clinical measure that would change with treatment in terms of exercise capacity or lung function in adults with severe lung disease as defined by an FEV1 < 40% predicted when clinically stable.

**Methods:**

10 adults homozygous for the Phe508del received LUM/IVA. We assessed; six minute walk test (6MWT), spirometry, gas transfer (DLCO), plethysmography, and nitrogen multiple breath washout (MBW) at baseline, 4, 12, 24 and 52 weeks. Comparison was made with 10 matched historical controls that had been observed over 12 months.

**Results:**

There was a significant improvement in 6MWT by 4 weeks of treatment; with a mean increase of 78 m (SD 62.3) and this increased to 118.1 m (SD 80.9) (ANOVA *p* = 0.006) by 52 weeks. Significant improvements were also seen in the resting heart rate and the oxygen saturation (SaO2) after 6 min walking. A significant improvement was not seen in FEV1 though until 24 weeks, though this was maintained at 52 weeks (ANOVA, *p* = 0.0004). There were no significant differences seen in the MBW or DLCO. After 12 months treatment with LUM/IVA, in comparison to historical controls; the 6MWT increased by 118 m (SD 80.9), but fell in the controls − 61.3 m (SD 31.1). FEV1; LUM/IVA led to an increase of 0.398 L/min, compared to a fall in the controls − 0.18 (SD 0.2).

**Conclusion:**

In adults homozygous for Phe508del with severe disease, treatment with LUM/IVA results in a clinically significant improvement in 6MWT that was evident at 4 weeks and maintained at 52 weeks. Improvement in exercise tolerance is an important outcome to consider in those with more severe airways disease.

**Trial registration:**

This was an observational trial conducted on individuals who became eligible to receive LUM/IVA. All investigations were carried out as part of routine clinical care. The trial was registered in retrospect on the 13/5/2019 on the Australian New Zealand Clinical Trials registry; ACTRN12619000708156.

## Background

Cystic Fibrosis (CF) is a genetic disorder caused by mutations in the gene that encodes for the Cystic Fibrosis Transmembrane Regulator (CFTR) protein, an epithelial ion channel that is crucial in regulating the flow of negatively charged ions across membranes and ensuring adequate hydration of mucus as well as influencing immune responses in ways that are incompletely understood [1]. As a consequence, dysfunction results in a multisystem disorder, that seriously impacts upon on the lungs leading to recurring airway infections, progressive airway wall damage, bronchiectasis, fibrosis and airflow obstruction, eventually leading to death or the need for lung transplantation. Lumacaftor/Ivacaftor (LUM/IVA) is a combination agent that targets CFTR. In the case of the most common CF mutation, Phe508del, Lumacaftor acts as a corrector of the dysfunctional protein allowing increased surface expression and Ivacaftor a potentiator, increasing function [2]. In patients older than 12 years, homozygous for the Phe508del mutation, and with a percent predicted (pp)FEV1 40–80%, treatment with LUM/IVA for 24 weeks led to a modest 2.6–4% increase in FEV1, however improvements were also seen in BMI and there was notably a 30–39% reduction in the pulmonary exacerbation rate [3]. Furthermore, assessment of this group after 96 weeks of treatment in comparison to matched registry controls, demonstrated a sustained impact with a reduction in the annual rate of decline in FEV1 [4].

The ability to extrapolate these findings to a more severe cohort with CF though is not clear. Further analysis of the original study cohort when stratified by lung function demonstrated that those with the most severe airflow obstruction showed a similar improvement in FEV1 compared to study participants with higher lung function [5]. Though it is unclear whether this small change in lung function would deliver a clinically meaningful benefit to subjects with more severe disease (< 40% ppFEV1). Finally, it is also unclear what clinical measure if any would be sensitive enough to detect a change, in those with advanced lung disease.

Our aim was to determine a sensitive clinical measure of change, in patients with CF and severe airways disease as measured by an FEV1 < 40% when stable, following treatment with LUM/IVA. We assessed response with spirometry and compared this to changes seen in exercise capacity as measured by the six minute walk test (6MWT), the nitrogen multi-breath washout test, the carbon monoxide gas transfer factor and lung volumes measured by plethysmography.

## Methods

We assessed the response in 10 adult participants from the John Hunter Hospital adult CF centre, aged ≥18 years, homozygous for Phe508del mutation, eligible for access to LUM/IVA through a compassionate access programme provided by Vertex. To be eligible they had to have an FEV1 ≤ 40% when clinically stable, or experienced a ≥ 20% fall in FEV1, or had been referred for lung transplantation assessment. Participants received Lumacaftor 400 mg and Ivacaftor 250 mg twice daily for 12 months.

LUM/IVA participants had the following assessments observed after accessing the compassionate access programme; spirometry, nitrogen MBW test and 6MWT performed on the day of commencing treatment, 4 weeks later, 12 weeks, 24 weeks and at 52 weeks. Plethysmography and single breath carbon monoxide gas transfer factor (DLCO) were performed at baseline, 24 weeks and 52 weeks.

These participants were compared to 10 adult historical controls, who attended the same centre from 2012 to 2015, aged ≥18 years, homozygous for the Phe508del mutation, who also would have been eligible for access to LUM/IVA as above, who either had ppFEV1 ≤ 40% when clinically stable at their annual review and or were judged to require referral for transplant at this time. Assessments were taken when clinically stable for their annual review, at baseline and again at the annual review approximately 12 months later. This included spirometry, plethysmography, DLCO and 6MWT.

Spirometry [6], DLCO [7], plethysmography [8] and 6MWT [9] were performed according to ATS/ERS standards. The Nitrogen MBW was performed using an EcoMedix Exhalyzer D running Spiroware Version 3.1.6 software. MBW technique adhered to the guidance of the American Thoracic Society (ATS) [8]. Only the LUM/IVA treated patients underwent MBW testing to determine lung clearance index (LCI) 2.5 functional residual capacity (FRC), sCOND (ventilation heterogeneity generated in the conductive lung zone) and sACIN (ventilation heterogeneity generated peripheral to the acinar entrance). The latter indices were analysed to determine if the region where ventilator inhomogeneity might be most impacted by LUM/IVA treatment could be demonstrated. All data for MBW was generated by the EcoMedix software.

Analysis of continuous variables was performed using parametric techniques to compare differences as these were normally distributed. This was done using a one-way ANOVA with Dunnett’s multiple comparisons test. Categorical comparisons were assessed using Fisher’s exact test. Analysis was carried out using Graphpad Prism version 7. The study was approved by the Hunter New England LHD Ethics committee. Subjects agreed to participate with written informed consent.

## Results

Participants are described in Table 1. Both groups were matched in terms of age, sex, the presence of diabetes requiring insulin therapy and exacerbations in the previous 12 months requiring treatment with intravenous antibiotics. Both groups also had severe airflow limitation and air trapping. They also demonstrated a mildly impaired 6MWT distance.

**Table 1:** Subject characteristics

	Received LUM/IVA	Not received LUM/IVA	
Number	10	10	
Age (range) years	26.5 (19–56)	30.6 (24–47)	*P* = 0.9
Sex M:F	6:4	5:5	
BMI	21.5 (3.3)	21.8 (3.0)	*P* = 0.8
FEV1% predicted	36.3 (2.8)	40.1 (6.6)	*P* = 0.1
Range	(32.4–40.1)	(32.1–44.3)	
FVC% predicted	66.4 (12.2)	66.5 (12.2)	P = 0.8
Range	(51.4–77.2)	(50.8–76.5)	
TLC (% predicted)	98.11 (16)	95.7 (1.4)	P = 0.8
FRC (% predicted)	122 (36.3)	124 (15.1)	P = 0.8
RV (% predicted)	200.4 (69.6)	198 (53)	*P* = 0.7
DLCO (% predicted)	58 (14.6)	63 (18.2)	*P* = 0.6
6 MWT m	532.4 (90.1)	546 (81.3)	P = 0.8
CF related diabetes	5	5	
Pseudomonas	9	9	
Burkholderia Cenocepacia	1	1	
Number of exacerbations in the previous 12 months requiring IV antibiotics	3 (4.6)	2.8 (5.6)	P = 0.8
Using regular nebulised DNase	10	10	NA
Using regular Azithromycin	8	9	P = 0.9
Using regular nebulised antibiotics	9	9	P = 0.8

**Fig. 1:**
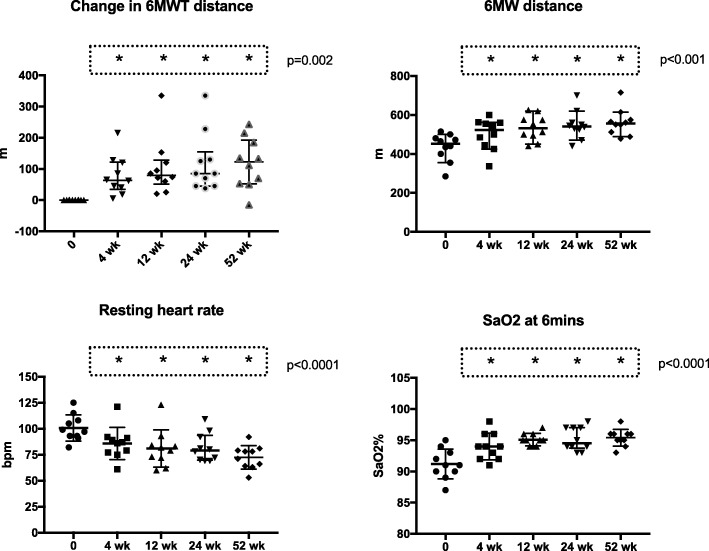
Data on the 6MWT. The data relates to the 10 subjects treated with LUM/IVA over 12 months. Individual data points are represented as well as the median and interquartile range. Analysis was done using one-way ANOVA with Dunnett’s multiple comparisons test. The overall ANOVA p value is given. The groups that are different from baseline (*p* < 0.05) are represented by *

In the 10 participants that received treatment with LUM/IVA, there was a significant improvement in exercise tolerance seen by 4 weeks. This was demonstrated by an increased change in 6MWT distance after 4 weeks of treatment; mean increase 74 m (SD 67.1) and this progressively increased by 12 months to 118.1 m (SD 80.9) (ANOVA *p* = 0.0018) (Fig. 1). A similar response was seen in total 6MWT distance (Fig. 1). There was a progressive decrease in resting or baseline heart rate at the commencement of the 6MWT, there was also an improvement seen in the fall in SaO2 after 6 min that was seen by 4 weeks and maintained at 52 weeks (Fig. 1).

**Fig. 2:**
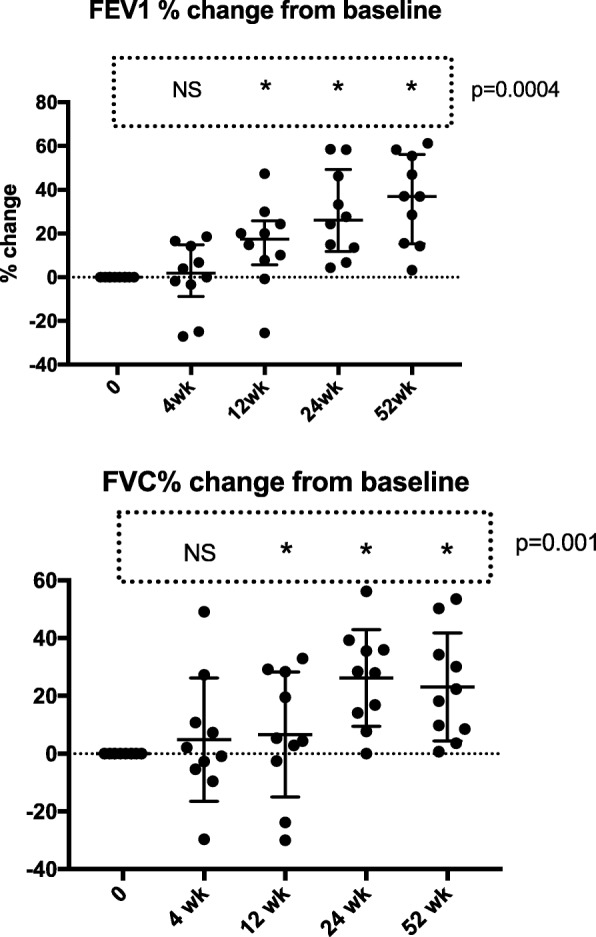
Data on FEV1 and FVC. The data relates to the 10 subjects treated with LUM/IVA over 12 months. Individual data points are represented as well as the median and interquartile range. Analysis was done using one-way ANOVA with Dunnett’s multiple comparisons test. The overall ANOVA *p* value is given. The groups that are different from baseline (*p* < 0.05) are represented by *

In comparison, a significant improvement was not seen in FEV1 until after 24 weeks, though this was maintained at 52 weeks (ANOVA, *p* = 0.0004). This represented a mean increase from baseline in FEV1 by 6 months of 28.8% (sd 20.1) and by 12 months 23.2% (SD 18.7) (Fig. 2). At 12 months this represents a relatively small, though significantly different absolute mean increase in FEV1, 0.398 L/min (0.34) from baseline (*p* < 0.001). Similarly, there was an increase in FVC (Fig.1), that became significant after 24 weeks; 26.2% (SD 16.8) and was maintained at 52 weeks 23.2% (SD 18.7) (ANOVA *p* = 0.001). This was a mean absolute increase of 0.492 L (SD 0.6) after 12 months.

**Fig. 3:**
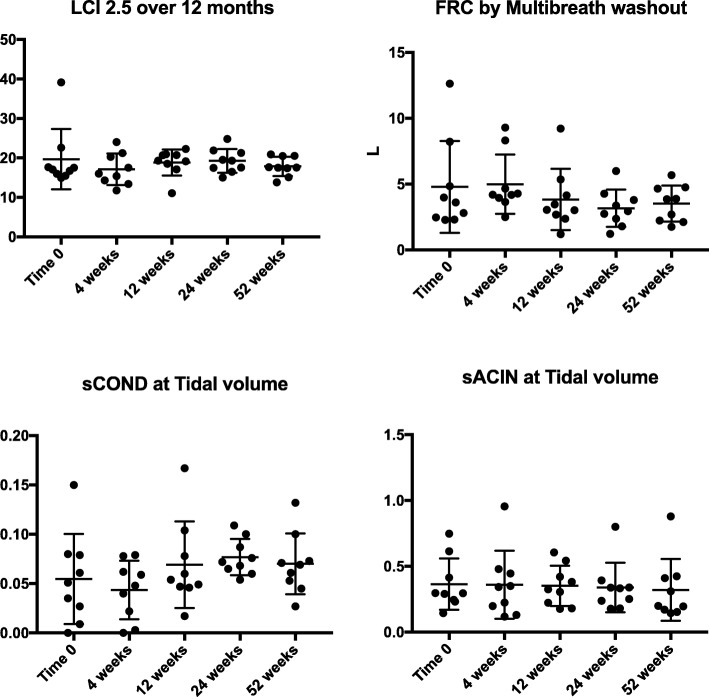
Data on the Nitrogen MBW. The data relates to the 10 subjects treated with LUM/IVA over 12 months. Individual data points are represented as well as the median and interquartile range. Analysis was done using one-way ANOVA with Dunnett’s multiple comparisons test. The overall ANOVA p value is given. No values are given for the y axis as these changes are without dimension

To determine if small changes in lung function could be discerned earlier than 24 weeks the nitrogen MBW was also performed and the lung clearance index (LCI 2.5), FRC, sCOND and sACIN measured at tidal volume breathing (Fig. 3) were derived. The LCI 2.5 was quite abnormal at baseline, mean 19.7 (SD 7.6). However, there was no significant difference seen in any of these parameters throughout the 12 months. Similarly, the DLCO was measured at the same time points, with no change seen (data not shown).

Adverse events following commencement of LUM/IVA were reported by 6/10 (60%). This included symptoms of chest tightness of dyspnoea, that was reported by all 6 within the first 24 h of commencing treatment. The symptoms persisted intermittently in 3/10 (30%). Headache was reported to be present intermittently in 2/10 (20%). In no cases were symptoms severe enough to warrant discontinuation.

**Fig. 4:**
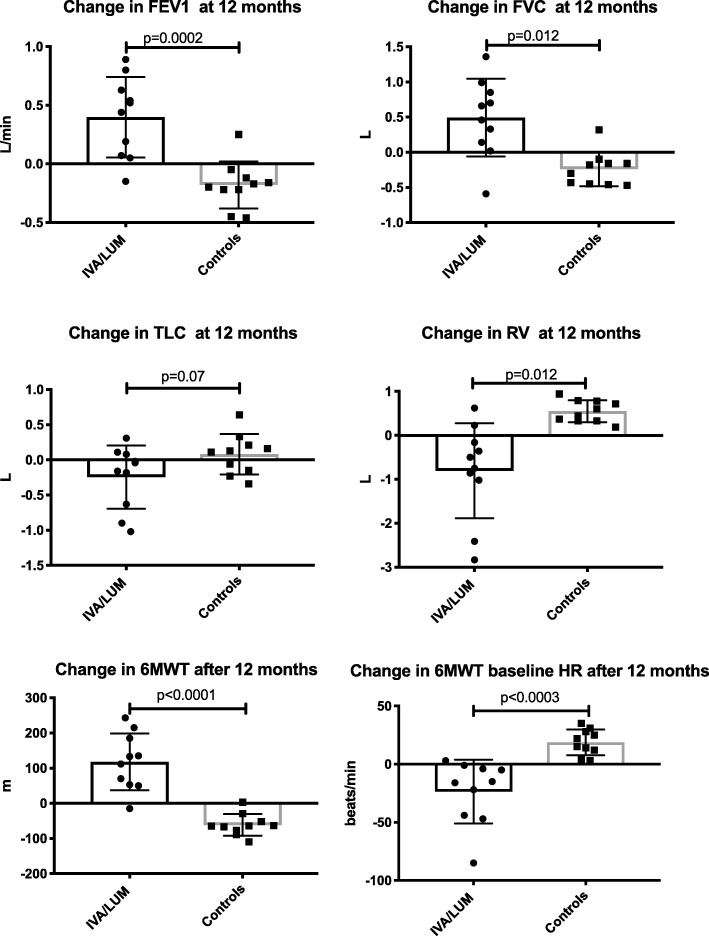
Comparison in the change seen over 12 months of treatment in 10 subjects treated with LUM/IVA. This is compared to the change seen in 10 age matched historical controls over a 12 month period of review. Data is represented as mean and standard deviation. Comparison made using Student’s T test

It is our centre’s usual practice to assess patients annually when they are clinically stable, with full lung function tests and in those with an FEV1 < 50% predicted, a 6MWT. We therefore had data from a similar cohort, homozygous for Phe508del, with severely impaired airflow obstruction that could be compared over a 12 month period and we used as matched historical controls to the group who had received LUM/IVA (Fig. 4). Exercise capacity was improved at 12 months in those receiving treatment. The 6MWT increased with LUM/IVA 118 m (SD 80.9) but decreased in the controls by − 61.3 m (SD 31.1). In the case of the controls the decrease in 6MWT may represent disease progression in those with severe airways disease. For FEV1; LUM/IVA led to an increase of 0.398 L/min, compared to a fall in the controls − 0.18 (SD 0.2). For FVC; an increase of 0.492 (SD 0.6) compared to a fall of − 0.24 L (0.2). Residual volume fell in those with IVA/LUM, − 0.8 L (SD 1.1), but rose in the controls by 0.55 L (SD 0.25). There was no difference in TLC or DLCO. The changes in lung function over 12 months were also associated with changes seen in the 6MWT distance; FEV1 r = 0.58 (*p* = 0.007), FVC r = 0.45 and RV r = − 0.64 (*p* = 0.02) (all, Pearson’s correlation coefficient). Finally, we compared exacerbations that had required treatment with intravenous antibiotics. Those treated with IVA/LUM had a mean number of 1.4 (SD 1.1) episodes in the 12 months, compared to 3.3 (SD 1.3) in the controls (*p* = 0.003).

## Discussion

The addition of LUM/IVA to the treatment regime of subjects homozygous for Phe508del with severe airflow obstruction led to a significant improvement in 6MWT distance after 4 weeks, which peaked at 24 weeks and was maintained at 52 weeks. Consistent with the effect seen in subjects with lesser degrees of airflow obstruction there was a small increase, both relative and absolute, in FEV1 and FVC seen by 24 weeks which was maintained at 52 weeks. There were no changes apparent in nitrogen MBW indices, this test was difficult to perform and insensitive to change in these subjects with severe lung disease. When compared to historical control subjects over 12 months LUM/IVA resulted in; improved exercise tolerance, improved FEV1, FVC and less air trapping as demonstrated by a fall in RV. The historical controls showed a small but significant deterioration in all these measures over an equivalent 12-month observation period.

While neither spirometry, nor MBW showed detectable change in the initial 4 weeks of LUM/IVA treatment, there was a significant improvement in exercise tolerance; an increase in 6MWT distance (mean increase = 74 m), along with improvements in resting heart rate and saturations after 6 min of walking. This effect of treatment was optimal by 6 months and maintained at 12 months (mean increase = 118 m). The improvement in 6MWT also correlated with the changes that were seen in lung function after 12 months observation. The improvement seen in 6MWT is clinically meaningful in this cohort with severe lung disease. An increase of between 14 and 30.5 m in the 6MWT has been determined to be the minimal clinically important distance across a wide range of cardiopulmonary disorders [10]. The value of 6MWT in CF though is mixed. In patients with mild to moderate airflow obstruction, the 6MWT distance was found not to be different from that of aged matched healthy controls and tests of maximal exercise capacity are recommended to detect the presence of early disease [11, 12]. However, in more severe CF disease, such as our population, the 6MWT has been used as a prognostic indicator for transplantation. For example, in 286 consecutive CF patients observed in a retrospective study, the 6MWT distance was shown to reliably correlate with FEV1 and a 6MWT distance of < 475 m with desaturation was found to be an independent predictor of death or need for transplant in patients with an ppFEV1 < 60% [13]. In addition, we are confident the changes that we saw represented a clinically important improvement, they were twice the minimal clinically important difference, sustained to 12 months and correlated closely with the improvement seen in lung function in particular air trapping after 12 months.

In comparison the magnitude of the change in lung function seen in the subjects with severe airflow obstruction was small in absolute terms and more variable, but in keeping with previous reports [3, 5]. A clear benefit of LUM/IVA did not become apparent until our subjects with severe lung disease completed 6 months of treatment; whereas it has been shown that in subjects with less severe disease (i.e. > 40% FEV1), to effect lung function within 14 days [3]. With only 10 subjects we were underpowered to determine a difference in lung function in light of the small change in FEV1 that was anticipated with LUM/IVA and the enhanced variability that could be expected with severe lung disease. Despite this with repeated measures and presumably a sustained effect on clinical outcomes, even with this relatively small sample we could see improved lung function by 24 weeks which was sustained at 52 weeks. In CF, airflow obstruction is an important marker of disease severity. A study of risk factors for death of patients with CF suggested that an FEV1 ≤ 30% predicted, hypercapnoea and the need for enteral nutritional supplements were all associated with a higher risk of death [14]. More recently a review of 3340 patients of the US CF data registry, showed that adults with an FEV1 < 30% predicted had a median survival of 6 years, with the risk of death increased if there was: a need to use oxygen, colonisation with B. cenocepacia, a BMI < 18, female sex, CF-related diabetes and 1 or more exacerbations in the previous year [15]. Participants in the study described herein did not have airflow obstruction ≤30%, were generally well nourished, but did have frequent exacerbations and half of the cohort had diabetes. After 1 year of LUM/IVA treatment none of the subjects had an FEV1 < 30%. In comparison 30% (3/10) of the control group, had an FEV1 that had decreased to < 30% after 12 months. By 12 months we could see that this improvement in FEV1 was associated with less air trapping and there also appeared to be fewer serious exacerbations requiring treatment with intravenous antibiotics, compared to the historical controls.

We speculated that in patients with severe airflow obstruction, spirometry change may not be sufficiently sensitive to demonstrate a measurable response to treatment. We therefore utilised nitrogen MBW to see if this technique would allow the earlier detection of changes in lung function, in particular ventilation inhomogeneity, with LUM/IVA. In MBW the derived parameter LCI2.5 (Lung clearance Index 2.5%) has been used as an index of ventilation inhomogeneity and has been shown to predict early lung disease in children and to correlate, at least in part, to the extent of change present on chest CT [16]. LCI2.5 is a unitless, derivative value calculated as the number of lung volume turnovers required to clear the lungs of the inert marker gas (i.e. nitrogen) to 1/40th of the starting concentration. We showed that in all cases of LUM/IVA subjects the LCI2.5 was substantially increased compared to predicted values and that there was no consistent improvement evident with LUM/IVA treatment. The MBW has also been used to detect the presence of small airways disease in asthma. Two indices of ventilation heterogeneity in peripheral airways are generated by the technique: one representing the inhomogeneity of ventilation in airways where gas transport is convection-dependent (sCOND) and the other the inhomogeneity of ventilation in more peripheral airways where gas movement becomes diffusion-dependent (sACIN). These measures have been associated in asthma with airway reactivity and neutrophilic inflammation [17]. Again however, LUM/IVA subjects did not demonstrate any improvement in either index. These data may suggest that in our LUM/IVA patients, all of whom had evidence of very severe airflow obstruction and extensive bronchiectasis, the ventilation inhomogeneity was too great and any sensitivity the MBW parameters may offer in the LUM/IVA-treated lung were lost with the noise and variability seen in this context. This suggestion is supported by the observation that abnormalities in first generation airways can interfere with Scond making anatomical differentiation imprecise. Further, the number of bronchial segments affected by bronchiectasis and LCI has been shown to have a dependent relationship that interferes with LCI [18, 19].

Adverse events have been reported with LUM/IVA, and Elborn et al. [5] reported more symptoms of dyspnoea and cough in patients having greater airflow obstruction i.e. ppFEV1 < 40%. Reports have also emerged of an initial fall in lung function in patients with ppFEV1 < 40% receiving LUM/IVA with up to 83% reporting symptoms of dyspnoea and/or chest tightness [20]. Poor tolerance of LUM/IVA would further limit any small clinical benefit that could be extrapolated from earlier studies to patients with more severe disease. We found in our group of patients that similar respiratory-related symptoms were common, though there were no serious adverse events and all participants were able to continue treatment. Given that such events occur more often with LUM/IVA in patients with more severe disease, caution should be exercised during the administration of LUM/IVA in this group of patients. However, if LUM/IVA treatment is tolerated the treatment is beneficial.

There are a number of limitations to our study. Our intervention group was observational and unblinded, as were investigators. They were also not compared to a prospective control arm and participants were not randomised to treatment. However, given the benefits now demonstrated by LUM/IVA in people homozygous with Phe508del with severe lung disease, and where treatment is already optimised such a study would probably be considered unethical and no reliable outcome measure is available in this group to predict response to treatment. However, our historical control group was selected from patients with similar disease severity, had received other similar long-term treatments and apart from treatment with LUM/IVA, were representative of patients with severe lung disease receiving optimal clinical management. Furthermore, the improvements seen in exercise tolerance were sustained and correlated with the small but significant improvement seen in lung function. For these reasons, the comparisons used here appears valid.

## Conclusions

In summary, we have demonstrated that in adults homozygous for Phe508del and with severe lung disease, treatment with LUM/IVA results in early and sustained improvements in the 6MWT and this is indicative of important clinically benefits. Improvement in our cohort was seen in spirometry and as anticipated these changes were small and variable and similar to that shown in the phase III clinical study [3].

Importantly, the 6MWT data shown herein suggests that this simple test provides a sensitive and early indicator of the functional impact of LUM/IVA treatment in CF patients with severe lung disease. It should therefore be considered more often in CF as an outcome measure in those with severe lung disease for both intervention trials, data registries and regulatory authorities to assess response to treatments.

## Data Availability

The data that support the findings of this study are available from Prof Peter Wark but restrictions apply to the availability of these data, that contain personal information and individuals may be identified. Deidentified data are however is available from the authors upon reasonable request and with permission of Hunter New England LHD Ethics committee.

## Abbreviations

6MWT: Six minute walk test
CF: Cystic Fibrosis
CFTR: Cystic Fibrosis Transmembrane Regulator
DLCO: Single breath carbon monoxide diffusion test
LUM/IVA: Lumcaftor/Ivacaftor
MBW: Nitrogen multiple breath washout
